# Body Fat Patterning, Hepatic Fat and Pancreatic Volume of Non-Obese Asian Indians with Type 2 Diabetes in North India: A Case-Control Study

**DOI:** 10.1371/journal.pone.0140447

**Published:** 2015-10-16

**Authors:** Anoop Misra, Shajith Anoop, Seema Gulati, Kalaivani Mani, Surya Prakash Bhatt, Ravindra Mohan Pandey

**Affiliations:** 1 Centre of Nutrition & Metabolic Research (C-NET), National Diabetes, Obesity and Cholesterol Foundation (N-DOC), SDA, New Delhi, India; 2 Diabetes Foundation (India), SDA, New Delhi, India; 3 Fortis C-DOC Centre of Excellence for Diabetes, Metabolic Diseases and Endocrinology, Chirag Enclave, Nehru place, New Delhi, India; 4 Fortis Flt. Lt. Rajan Dhall Hospital, Vasant Kunj, New Delhi, India; 5 Department of Biostatistics, All India Institute of Medical Sciences, New Delhi, India; Bambino Gesù Children's Hospital, ITALY

## Abstract

**Objective:**

To evaluate body fat patterning and phenotype including hepatic fat and pancreatic volume of non-obese (BMI: < 25 kg/m^2^) Asian Indians with type 2 diabetes residing in North India.

**Methods:**

Non-obese patients with type 2 diabetes (*n* = 93) and non-obese, normo-glycemic subjects (*n* = 40) were recruited. BMI, waist & hip circumferences, skinfold thickness at 8 sites, body fat, lean mass and detailed abdominal fat evaluation [total abdominal fat, total subcutaneous fat (superficial, deep, anterior, and posterior), total intra-abdominal fat (intra-peritoneal, retroperitoneal)], liver span, grades of fatty liver and pancreatic volume were compared.

**Results:**

Waist circumference, subscapular skinfolds and total truncal fat (on DEXA) were higher whereas calf, total peripheral skinfolds and total leg fat (on DEXA) lower in patients. Specifically, the following volumes were higher in cases as compared to controls; total abdominal fat (19.4%), total intra-abdominal fat (49.7%), intra-peritoneal fat (47.7%), retroperitoneal fat (70.7%), pancreatic volume (26.6%), pancreatic volume index (21.3%) and liver span (10.8%). In cases, significant positive correlations were observed for pancreatic volume with BMI, waist and hip circumferences, W-HR, subscapular, abdominal and total truncal skinfolds, truncal, total subcutaneous, total intra-abdominal, intra-peritoneal, retroperitoneal fat depots, liver span and fatty liver.

**Conclusions:**

In non-obese Asian Indians with type 2 diabetes, subcutaneous and intra-abdominal obesity, including fatty liver, and pancreatic volume were higher and peripheral subcutaneous adiposity was lower than BMI matched non-diabetic subjects. Importantly, increased pancreatic volume in patients was highly correlated with multiple measures of abdominal obesity and liver fat.

## Introduction

The prevalence of type 2 diabetes is high and continues to increase in India [1]. Globally, a rising trend in type 2 diabetes is witnessed in young adults, including Asians [2], usually related to obesity. However, even non-obese Asian Indians develop insulin resistance at an early age with a risk of type 2 diabetes and cardiovascular diseases [3].This feature has been attributed to genetic susceptibility [4], foetal programming [5] and low birth weight [6].

As compared with the White Caucasians, the characteristic phenotypic features of Asian Indians *viz;* higher body fat, excess truncal fat and lower lean body mass [7], are important contributors to insulin resistance, metabolic syndrome and development of type 2 diabetes [8]. In particular, abdominal obesity and thick subcutaneous adipose tissue are common in South Asians, and are evident even in non-obese people [9].

The pathogenesis of type 2 diabetes in non-obese Asian Indians [body mass index (BMI) < 25 kg/m^2^] is ill-understood and the relative contributions of insulin secretion and insulin resistance continue to be debated. Specifically, it remains unanswered if ‘non-obese’ Asian Indians have high total body fat and abdominal fat despite BMI being in the non-obese range which could contribute to insulin resistance.. Further, there is paucity of data regarding liver fat and pancreatic anatomy (e.g. volume, fat content) in relation to type 2 diabetes in Asian Indians.

The accurate quantification of body fat by robust and a non-invasive technique is of prime importance for clarifying pathophysiology and for rational clinical management (e.g. use of metformin) of type 2 diabetes. Previously, our group has carried out magnetic resonance imaging (MRI) of abdominal fat in non-diabetic Asian Indians to quantify various abdominal fat compartments [10,11], however patients with type 2 diabetes were not studied. Further, there are no reports on liver fat and pancreatic volume in Asian Indians.

In the present study on non-obese (BMI < 25 kg/m^2^) Asian Indians with type 2 diabetes, we intended to describe detailed body composition data specifically dealing with truncal fat, abdominal fat, and some data on liver and pancreas, by skinfold measurements, Dual Energy X ray Absorptiometry (DEXA) scan and detailed MRI studies.

## Methodology

This study was approved by the institutional ethics committee and conducted at the outpatient department of Fortis hospital, New Delhi, India. Non-obese (BMI < 25 kg/m^2^) patients with type 2 diabetes, diagnosed within one year from onset (cases, *n* = 93) and BMI-matched, non-diabetic subjects (controls, *n* = 40), aged between 18–40 years, were recruited after obtaining informed and written consent. Pregnant and lactating women, subjects having ketonuria, on insulin therapy, or on drugs known to affect body composition (steroids or thiazolidinediones),with history of significant alcohol intake, subjects with metallic implants, pacemaker leads, radioactive seeds or surgical staples in the body were excluded for the study.

Anthropometric measures were recorded as mentioned previously [12]. Skinfold measurement was done using a Lange skinfold calliper (Beta Technology Inc., Santa Cruz, CA, USA). Peripheral skinfolds (biceps, triceps, thigh and calf skinfolds; sum = total peripheral skinfolds) and truncal skinfolds [subscapular, supra iliac & abdominal skinfolds (diagonal & vertical; sum = total truncal skinfolds] were measured as reported previously [13].

Blood samples were collected after an overnight fast and postprandial blood samples were collected after a standardised meal of 250 calories. Biochemical analysis was performed according to methods described previously [8].

### DEXA Scan

Whole body DEXA scans were performed using a LUNAR Prodigy Advance DEXA machine (G.E. Medical Systems, Madison, WI, USA).The DEXA scanner was calibrated every day for precision by scanning an aluminium spine phantom. Bilateral sections and the whole body were obtained by analysis of the DEXA scans using the Prodigy encore software (Version 12.30.2008).The regions of interest (ROI) were marked using cut points on the image of the whole body scan.

### Abdominal Fat Quantification, Pancreas & Liver Imaging

Abdominal fat depots, liver span, grades of fatty liver and pancreatic volume were imaged using MRI (1.5 Tesla Signa HDxt, GE Health-care, Waukesha, USA) and quantified by ROI analysis using GE Advantage Workstation Volume Viewer software. Total abdominal fat & abdominal fat compartments *viz*: subcutaneous fat (anterior, posterior, superficial & deep) and intra-abdominal fat (intra-peritoneal & retroperitoneal) were identified using previously published protocol [14] & quantified by T1 weighted axial scans between L2/L3 of the lumbar vertebrae, using a defined MR imaging protocol (S1 Table). These regions were further analysed by manually drawing the ROI on the T1 weighted axial slice mentioned above.

Liver span was measured using a T2 weighted coronal scan as per protocol (S1 Table).The field of view (FOV) spanning the whole liver was chosen. ROI analysis was performed on the slice displaying the maximum span of the liver and the distance was measured. Fat infiltration in the liver was measured using IN/OUT FSPGR sequence. Two images (one; in-phase and one; out of phase) were obtained for each slice. Hepatic fat infiltration was estimated by measuring the signal differences between the two images and graded as nil, grade 1, grade 2 or grade 3 [15]. Pancreatic volume was measured using 3D LAVA pulse sequence in accordance to the protocol (S1 Table). Pancreatic volume index was calculated as pancreatic volume (cm^3^) / body surface area (m^2^) [16].

## Statistical Analysis

Data were managed on an Excel spreadsheet (Microsoft Corp, Washington, USA), analysed using STATA 11.0 (College station, Texas, USA) and presented as frequency (percentage values) or mean ± standard deviation / median (min—max) as appropriate. Analysis of covariance was used to adjust for imbalances in age between cases and controls. Categorical variables were analysed using Chi square test. The correlation of anthropometric, abdominal adiposity measures and liver span with pancreatic volume was assessed using Pearson’s correlation. The differences in median values of abdominal fat and liver span among quartiles of pancreatic volume were tested using Kruskal-Wallis test followed by Bonferronni correction. The *p* value < 0.05 was considered statistically significant.

## Results

In cases (males: *n* = 83, females: *n* = 10), the mean age (36.3 ± 5.1) was significantly higher (*p* < 0.01) as compared to controls (males: *n* = 24, females: *n* = 16; Mean age: 27.8 ± 4.8 years). At similar BMI values, significantly higher mean values were observed in cases for age, waist circumference and W-HR (Table 1). Among males, the mean waist circumference (≥ 90 cms) was higher in cases (*n* = 60; 72.2%) as compared to controls (*n* = 5; 12.5%) while in females, waist circumference (≥ 80cms) was higher in controls (*n* = 12; 75%) as compared to cases (*n* = 5; 50%).

**Table 1:** Anthropometric profile.

Anthropometric variables	Unadjusted for age			Adjusted for age		
	Cases (n = 93)	Controls (n = 40)	*p value*	Cases (n = 93)	Controls (n = 40)	*p* value
Body mass Index (kg/m2)	22.8 ± 2.0	22.3 ± 2.1	0.21	22.8± 1.9	22.4 ± 1.8	0.33
Waist circumference (cms)	22.8 ± 2.1	82.8 ± 7.7	< 0.01*	85.5± 6.7	83.2 ± 6.9	0.09
Hipc ircumference (cms)	22.8 ± 2.2	90.8 ± 7.3	0.23	89.3± 4.8	91.3± 5.0	0.06
Waist to hip ratio	22.8 ± 2.3	0.90 ± 0.0	< 0.001*	0.95± 0.0	0.90 ± 0.0	< 0.001*
Mid arm Circumference (cms)	22.8 ± 2.4	27.6 ± 5.4	0.6	27.2 ±2.8	27.6 ± 3.1	0.53
Mid-thigh circumference (cms)	22.8 ± 2.5	48.7± 4.8	0.24	49.7± 3.8	48.7 ± 4.4	0.26

Values are presented as Mean ± SD,

*p < 0.05: Statistically significant.

**Fig 1:**
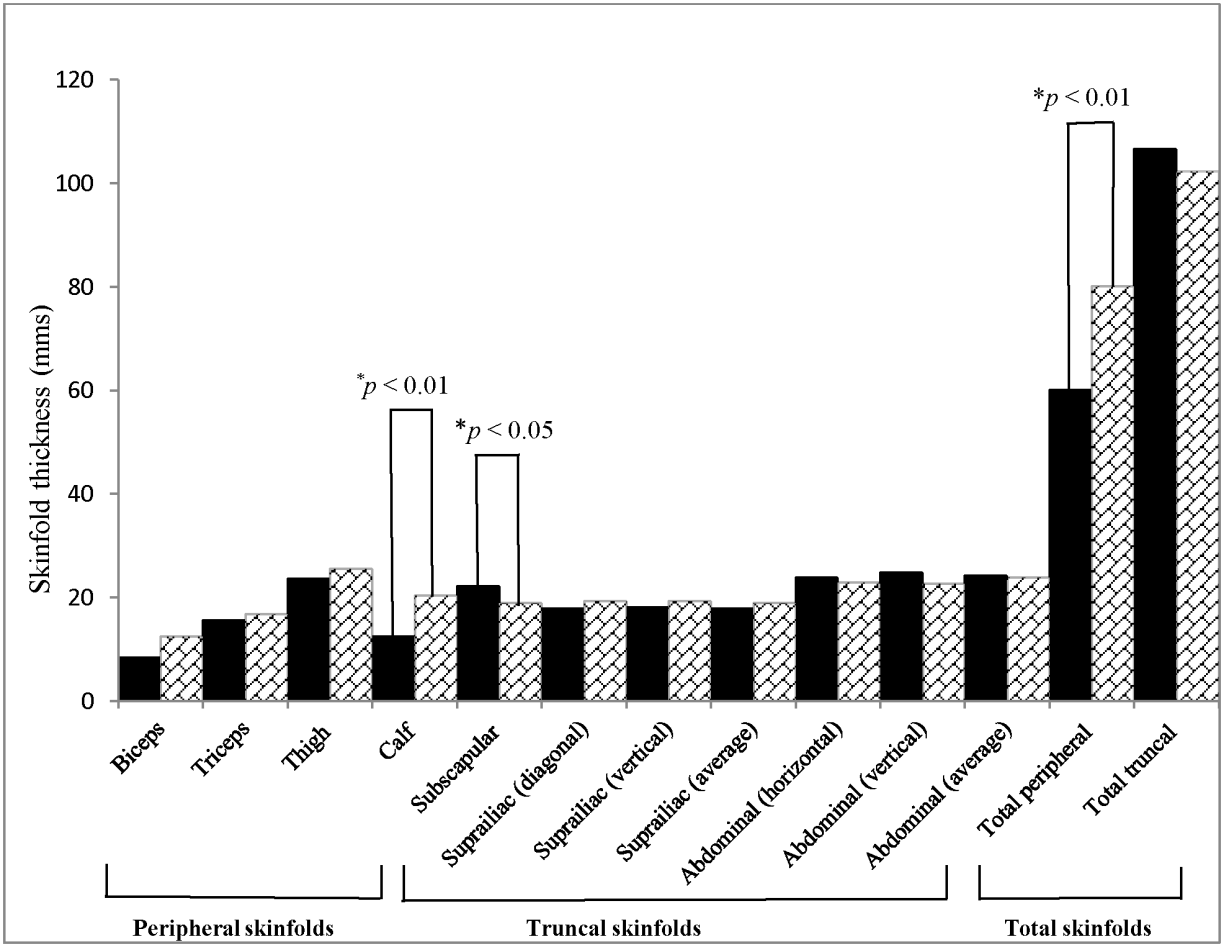
Skinfold measurements in cases (*n* = 93, shown in black bars) *vs*. controls (*n* = 40, shown in bars filled with crossed lines).

In cases, the mean values of subscapular skinfold thickness were significantly higher, whereas the mean values of calf and total peripheral skinfold thickness were lower as compared to controls. On adjustment for age, significant differences were observed between cases and controls, for waist to hip ratio, supra iliac skinfolds (horizontal & vertical) and peripheral skinfolds *viz*: biceps, triceps, thigh and calf skinfolds (Fig 1, S2 Table).

On DEXA, the mean values were significantly higher for total lean mass, total fat free mass, truncal fat mass and truncal lean mass in cases as compared to controls. The mean values of total leg fat %, total leg fat mass, including fat % and fat mass in right and left legs were significantly higher in controls as compared to cases, even after adjusting for age (S3 Table).

**Fig 2:**
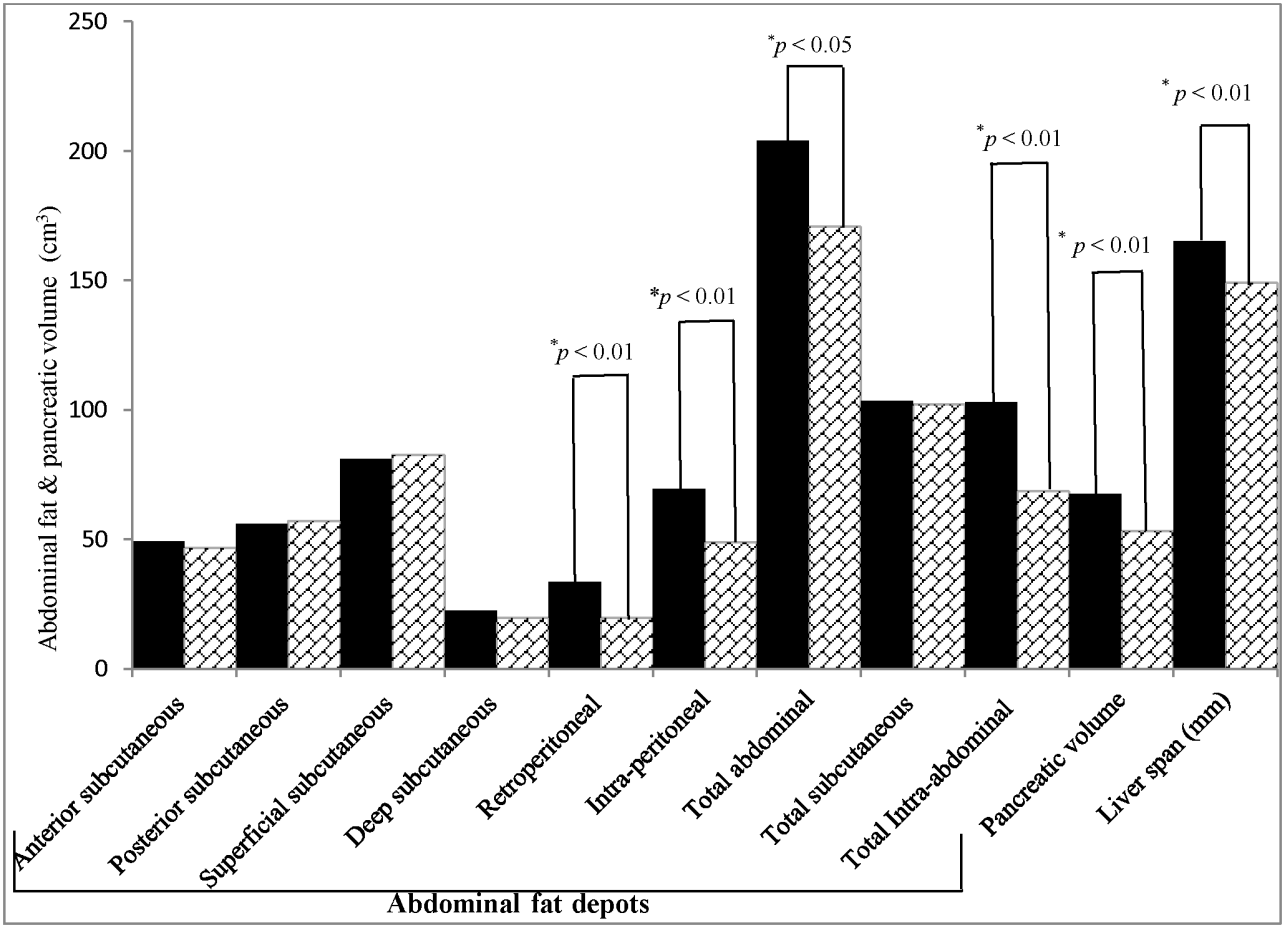
Abdominal fat depots, pancreatic volume & liver span measured by MRI (1.5 Tesla) in cases (*n* = 93, shown in black bars) & controls (*n* = 40, shown in box filled with crossed lines).

**Fig 3:**
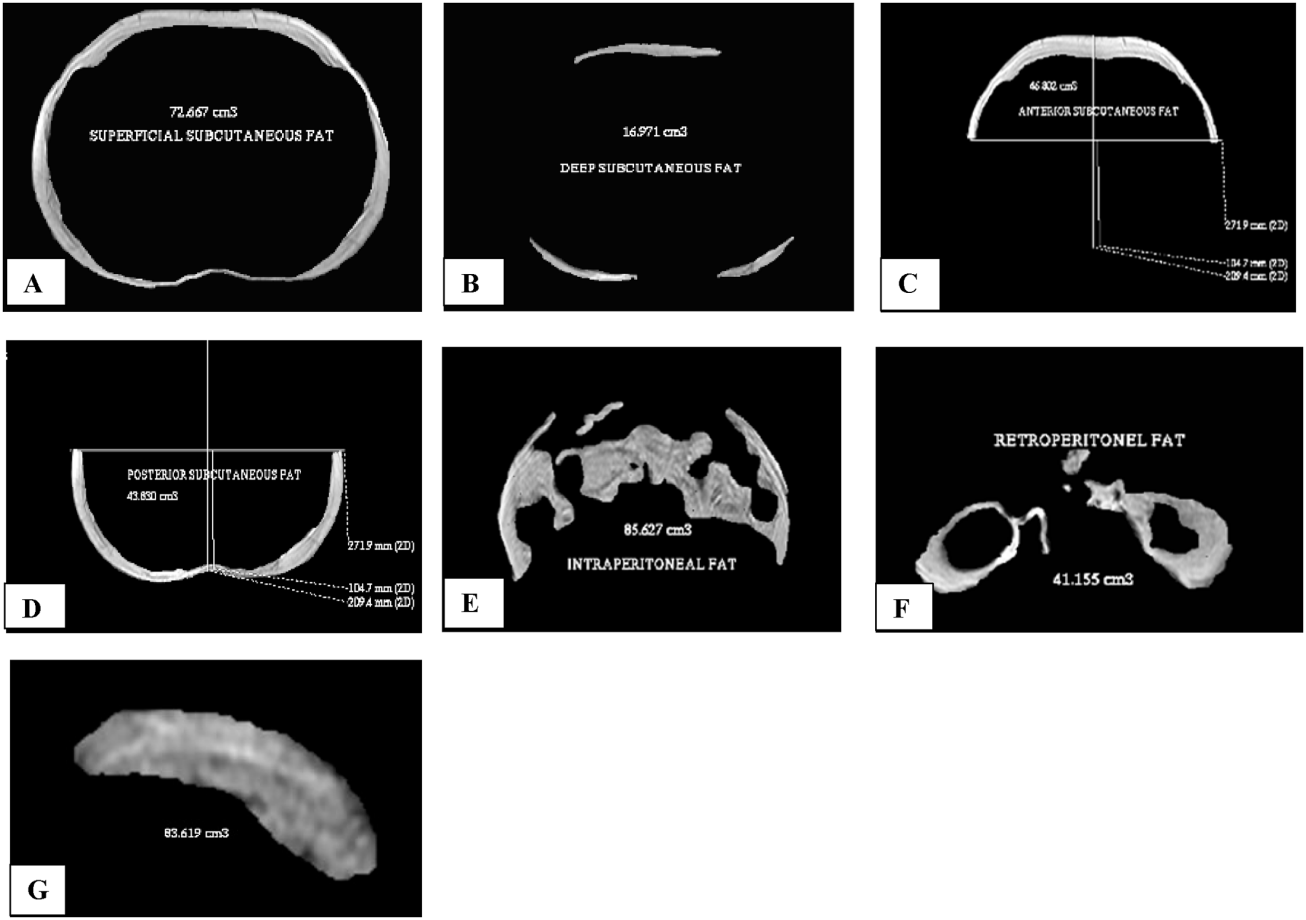
Abdominal fat depots and pancreatic volume in a 33 year old non-obese patient with type 2 diabetes, quantified from image obtained using MRI (1.5 Tesla) at L2/L3 region of lumbar vertebrae (A): Superficial abdominal subcutaneous fat, (B): Deep abdominal subcutaneous fat, (C): Anterior abdominal subcutaneous fat, (D): Posterior abdominal subcutaneous fat, (E): Intra-peritoneal fat, (F): Retroperitoneal fat & (G): Pancreatic volume.

Volumes of abdominal fat measures were significantly higher in cases as compared to controls; total abdominal fat (19.4%; *p* < 0.05), total intra-abdominal fat (49.7%; *p<* 0.001), intra-peritoneal fat (47.7%; *p<* 0.001) and retroperitoneal fat (70.7%; *p<*0.001). Further, in cases, significantly higher mean values were observed for pancreatic volume (26.6%; *p <* 0.001), pancreatic volume index (21.3%; *p<* 0.01) & liver span (10.8%; *p <* 0.001), as compared to controls, even after adjustment for age (Figs 2 and 3, S4 Table).

Fatty liver was observed in 48 cases (51.6%; 36 cases had grade 1 and 12 cases had grade 2 fatty liver) and 2 controls (5%; grade 1 fatty liver). Liver span correlated significantly (*p<*0.05) with grades of fatty liver.

In cases, pancreatic volume showed significant positive correlation with age, BMI, waist circumference, hip circumference, W-HR, abdominal skinfolds, total truncal skinfolds, truncal fat percentage, total subcutaneous fat, total abdominal fat, total intra-abdominal fat, intra-peritoneal fat, retroperitoneal fat, and liver span. In controls, significant positive correlations were observed for W-HR, abdominal skinfolds, total intra-abdominal fat, intra-peritoneal, and retroperitoneal fat (Table 2).

**Table 2:** Correlations of anthropometry, body composition, abdominal fat compartments and liver span with pancreatic volume.

Variables	Cases (n = 93)	*p* value	Controls (n = 40)	*p* value
	Pearsons's correlation coefficient (*r*)		Pearsons's correlation coefficient (*r*)	
Age (yrs)	0.26	< 0.05*	0.17	0.29
Body mass index (kg/m2)	0.34	<0.001*	0.27	0.08
Waist circumference (cms)	0.44	<0.001*	0.12	0.52
Hip circumference (cms)	0.42	<0.001*	-0.15	0.44
Waist—hip ratio	0.23	<0.05*	0.38	< 0.05*
Mid- arm circumference (cms)	0.4	<0.001*	0.21	0.28
Mid-thigh circumference (cms)	0.41	<0.001*	0.11	0.58
Sub scapular skinfolds (mms)	0.21	<0.05*	0.23	0.23
Supra iliac skinfolds (horizontal) (mms)	0.16	0.12	0.14	0.48
Supra iliac skinfolds (vertical) (mms)	0.13	0.19	0.34	0.09
Abdominal skinfolds (horizontal) (mms)	0.34	< 0.001*	0.45	< 0.05*
Abdominal skinfold (vertical) (mms)	0.37	< 0.001*	0.48	< 0.05*
Average abdominal skinfolds (mms)#	0.29	< 0.01*	0.07	0.7
Thigh skinfolds (mms)	0.05	0.59	0.08	0.68
Calf skinfolds (mms)	0.11	0.26	0.08	0.65
Total peripheral skinfolds (mms)*	0.06	0.52	-0.09	0.58
Total truncal skinfolds (mms)*	0.3	< 0.01*	0.07	0.64
Fasting blood glucose (mg/dl)	0.42	0.96	0.35	0.84
Post prandial blood glucose (mg/dl)	0.16	0.11	0.25	0.13
Glycosylated haemoglobin (%)	0.03	0.73	0.2	0.24
Serum glutamic pyruvic transaminase (U/L)	0.15	0.12	0.21	0.21
Serum glutamic oxaloacetic transaminase (U/L)	0.05	0.61	0.23	0.17
Total cholesterol (mg/dl)	0	0.16	0.16	0.35
High-density lipoprotein cholesterol (mg/dl)	-0.32	< 0.01*	-0.22	0.24
Low-density lipoprotein cholesterol (mg/dl)	0.12	0.22	0.24	0.15
Total body fat % ǂ	0.14	0.18	-0.06	0.71
Truncal fat mass (kg)ǂ	0.36	< 0.001*	0.32	<0.05*
Total abdominal fat volume (cm3)€	0.2	< 0.05*	0.06	0.72
Anterior subcutaneous fat volume (cm3)€	0.17	0.09	0.08	0.59
Posterior subcutaneous fat volume (cm3)€	0.14	0.17	0.07	0.65
Superficial subcutaneous fat volume (cm3)€	0.14	0.15	0.06	0.67
Deep subcutaneous fat volume (cm3)€	0.28	<0.01*	0.16	0.31
Total subcutaneous fat volume (cm3)€	0.2	<0.05*	0.09	0.57
Retroperitoneal fat volume (cm3)€	0.47	<0.01*	0.45	< 0.01*
Intra-peritoneal fat volume (cm3)€	0.32	<0.01*	0.47	< 0.01*
Total intra-abdominal fat volume (cm3)€	0.34	< 0.001*	0.48	< 0.01*
Liver span (mm)€	0.21	<0.05*	-0.05	0.75

* *p*< 0.05: Statistically significant.

*Total peripheral skinfolds = Sum of biceps, triceps, thigh & calf skinfolds,

*Total truncal skinfolds = Sum of sub scapular, suprailiac (horizontal & vertical) & abdominal skinfolds (diagonal & vertical).

^#^Average abdominal skinfolds = Mean of abdominal skinfolds (vertical+ horizontal).

^ǂ^ Measured by Dual energy X ray absorptiometry.(see text for details).

^**€**^ Measured by MRI (1.5 Tesla) at L2/L3 region of the lumbar vertebrae (see text for details).

Among biochemical variables, significantly, higher values were observed for; glycaemic parameters (fasting, postprandial blood glucose and glycosylated haemoglobin), lipids and hepatic transaminases in cases, as compared to controls, even after adjustment for age (S5 Table). According to the IDF definition [17], metabolic syndrome was recorded in 30 cases (32.2%) & 5 controls (12.5%). Further, significant negative correlation with pancreatic volume (*p* < 0.01) was observed only for HDL in cases while no significant correlation was observed for levels of glycosylated haemoglobin, fasting and postprandial blood glucose and hepatic transaminases, as compared to controls

Across quartiles of pancreatic volume, in cases, significant positive association was observed for total intra-abdominal fat, intra-peritoneal fat and retroperitoneal fat. Significant associations were observed between first and third quartile, first & fourth quartile, between second and third quartile and between third and fourth quartiles of total intra-abdominal fat, retroperitoneal fat and intra-peritoneal fat. In controls, significant associations were observed between the first and fourth quartiles and between second and fourth quartiles for total intra-abdominal fat, intra-peritoneal fat and retroperitoneal fat (S6 Table, Sl Fig). Pancreatic volume correlated significantly (*p* < 0.05) with liver span in patients with fatty liver as compared to those without fatty liver (S2 Fig).

## Discussion

This case-control study shows that relatively young, non-obese Asian Indians with type 2 diabetes have excess adiposity of all abdominal fat compartments, increased pancreatic volume and liver span and have decreased fat, specifically subcutaneous fat in limbs & calf, even after adjustment for age. This characteristic body composition has been reported for the first time in Asian Indians and is of importance for the pathophysiology as well as for treatment of diabetes in non-obese Asian Indians.

We have previously carried out several studies to define abdominal fat compartments in Asian Indians and their relation with cardiovascular risk factors and metabolic syndrome. These studies, done mostly in non-diabetic subjects showed strong correlation of truncal subcutaneous fat with metabolic syndrome ([10,11]. These previous findings are in partial variance with findings of the present study which clearly show that overall abdominal adiposity and intra-abdominal adiposity, and not only truncal or abdominal subcutaneous fat, are higher in patients with type 2 diabetes who are ‘non-obese’ as defined by BMI.

The findings of increased abdominal adiposity with increased intra-abdominal fat, liver span, pancreatic volume and pancreatic volume index hold significance to dysmetabolic state in Asian-Indians. Increased abdominal adiposity and large adipocyte size in Asian Indians [18] correlate with increased release of non-esterified free fatty acids (NEFAs) as compared to Whites [19]. Importantly, NEFA overload in islet cells of the pancreas leads to impaired beta cell response to hyperglycemia, effects that are partially termed as ‘lipotoxicity’. Prolonged hyperglycemia and lipotoxicity may result in fatty deposit and deranged functions of pancreatic islet cells, similar to the effects of steato-necrosis in the liver [20, 21]. Interestingly, we have also shown that there is significant negative correlation of pancreatic volume with high-density lipoprotein cholesterol (HDL). Specifically, high-density lipoprotein cholesterol enhances pancreatic beta cell function and inhibits apoptosis [22]. In type 2 diabetes, there is marked dyslipidemia, overriding the protective effects of HDL, eventually leading to fat infiltration in the pancreas and beta cell apoptosis.

In this study, we have considered liver span as surrogate marker of fatty liver and shown that higher grades of fatty liver are highly correlated with increased span. We also show that intra-abdominal fat and pancreatic volume correlate positively with liver span and the latter to fatty liver. Importantly, fatty liver is central to the development of insulin resistance and metabolic syndrome [23]. In particular, Asian Indians with fatty liver are highly predisposed to develop type 2 diabetes [12]. Further, hepatic triglyceride accumulation is significantly higher in Asians Indians *vs*. BMI matched Whites, and is associated with higher insulin resistance in the former [24]. We have previously shown, using proton magnetic resonance spectroscopy, that non-obese, non-diabetic individuals with fatty liver have deranged gluconeogenesis [25]. The cause of increased deposition of triglycerides in liver of Asian Indians is unknown, however, as previously stated; NEFA influx into the liver *via* the hepatic portal vein, due to increased abdominal adiposity [26] may contribute to this feature. This may occur in the background of genetic predisposition to develop fatty liver in Asian Indians as previously shown by us [27].

As compared to lean Whites, lean Asian Indians have thicker truncal skinfolds associated with insulin resistance [28] signifying that thicker truncal subcutaneous fat hold metabolic significance specifically in Asian Indians. We observed significant positive correlation between abdominal skinfold thickness and pancreatic volume in cases as compared to controls. The significance of lower peripheral skinfold thickness in patients with type 2 diabetes as compared to non-diabetic individuals in the current study is not entirely clear, however, it may mean less metabolic significance of this adipose tissue depot with regard to hyperglycemia in this ethnic group.

Technologically, it is difficult to define pancreatic anatomy, specifically fat infiltration, and research in this area is sparse. Certain studies have shown that patients with type 1 diabetes have lower pancreatic volume than patients with type 2 diabetes [17]. Pancreatic volume in patients with type 2 diabetes either have been shown to be low [29], or not correlated with diabetes [30] in other ethnic groups. In this context, increased pancreatic volume and its close correlation to hepatic and abdominal fat in the current study on non-obese Asian Indians with type 2 diabetes is an important yet previously unreported finding. It is possible that patients with shorter duration of type 2 diabetes, as in the present study, may have higher pancreatic volume than in those with longer duration, where progressive death of beta cells and consequent fibrosis may cause ‘pancreatic shrinking’. It is also possible that Asian Indians have increased tendency of fatty infiltration of pancreas (similar to fatty liver) which may cause increase in pancreatic volume. Progressive beta-cell dysfunction may be attributed to pancreatic fat accumulation in Asian Indians. Importantly, non-invasive measurement of pancreatic triglyceride concentration with magnetic resonance spectroscopy (MRS) in animals and human beings showed gradually increasing amount of fat infiltration from normoglycemia, prediabetes to diabetes [31]. Although we could not measure pancreatic function or quantify fat infiltration in the pancreas with the current MRI technique, increased pancreatic volume, similar to increased liver span, may mean increased pancreatic fat. In type 2 diabetes patients with increased pancreatic volume, quantification of pancreatic fat and its relation with beta cell dysfunction needs further research, especially in Asian Indians.

In summary, in this study we have defined body composition of young, non-obese patients with recently diagnosed type 2 diabetes, clearly showing that they feature excess adiposity in trunk and abdomen, and less subcutaneous fat in peripheral regions of the body. In addition we also show increased liver fat in nearly half of patients. Specifically, increased pancreatic volume in these patients may indicate fatty pancreas, correlating with other adiposity measures. Finally, these ‘non-obese’ patients are significantly obese centrally, which may result in heightened insulin resistance, and in addition may have pancreatic dysfunction due to fatty pancreas. This study has important relevance for further research on pancreatic anatomy and functions, pathophysiology of diabetes and also for use of appropriate therapy (e.g. metformin) in Asian Indians.

## Supporting Information

S1 Fig Box plots showing significant association of intra-peritoneal fat across quartiles of pancreatic volume in cases (A) & controls (B). (TIFF)

S2 Fig Scatter plots showing correlation of pancreatic volume with liver span in patients with fatty liver (A) *vs*. those without fatty liver (B). (TIFF)

S1 Table Technical protocol for quantification of abdominal fat, liver span, liver fat and pancreatic volume using MRI (1.5) Tesla at L2/L3 lumbar vertebrae. (DOCX)

S2 Table Comparison of skinfold measurement at eight sites. (DOCX)

S3 Table Body composition as measured by Dual energy X ray absorptiometry. (DOCX)

S4 Table Abdominal fat depots, liver span and pancreatic volume, quantified from image obtained by magnetic resonance imaging (1.5 Tesla) at L2/L3 region of the lumbar vertebrae. (DOCX)

S5 Table Biochemical profile. (DOCX)

S6 Table Association of abdominal fat depots and liver span across quartiles of pancreatic volume. (DOCX)
