# Correction: Body Fat Patterning, Hepatic Fat and Pancreatic Volume of Non-Obese Asian Indians with Type 2 Diabetes in North India: A Case-Control Study

**DOI:** 10.1371/journal.pone.0142749

**Published:** 2015-11-05

**Authors:** Anoop Misra, Shajith Anoop, Seema Gulati, Kalaivani Mani, Surya Prakash Bhatt, Ravindra Mohan Pandey

There is an error in Table 1. The values in the “Cases (n = 93)” column in the “Unadjusted for age” section of the table are incorrect. Please see the corrected Table 1 here.

**Table 1:** Anthropometric profile

Anthropometric variables	Unadjusted for age			Adjusted for age		
	Cases (*n* = 93)	Controls (*n* = 40)	*p* value	Cases (*n* = 93)	Controls (*n* = 40)	*p* value
Body mass Index (kg/m^2^)	22.8 ± 2.0	22.3 ± 2.1	0.21	22.8± 1.9	22.4 ± 1.8	0.33
Waist circumference (cms)	85.7 ± 6.8	82.8 ± 7.7	< 0.01*	85.5± 6.7	83.2 ± 6.9	0.09
Hip circumference (cms)	89.5 ± 4.6	90.8 ± 7.3	0.23	89.3± 4.8	91.3± 5.0	0.06
Waist to hip ratio	0.95 ± 0.0	0.90 ± 0.0	< 0.001*	0.95± 0.0	0.90 ± 0.0	< 0.001*
Mid arm circumference (cms)	27.2 ± 2.4	27.6 ± 5.4	0.60	27.2 ±2.8	27.6 ± 3.1	0.53
Mid-thigh circumference (cms)	49.7 ± 4.2	48.7± 4.8	0.24	49.7± 3.8	48.7 ± 4.4	0.26

Values are presented as Mean ± SD,

*p < 0.05: Statistically significant
