# Supplementary figures and images for: Body Fat Patterning, Hepatic Fat and Pancreatic Volume of Non-Obese Asian Indians with Type 2 Diabetes in North India: A Case-Control Study
Source: PLoS One. 2015 Oct 16;10(10):e0140447. doi: 10.1371/journal.pone.0140447 (PMC4608569; doi:10.1371/journal.pone.0140447)

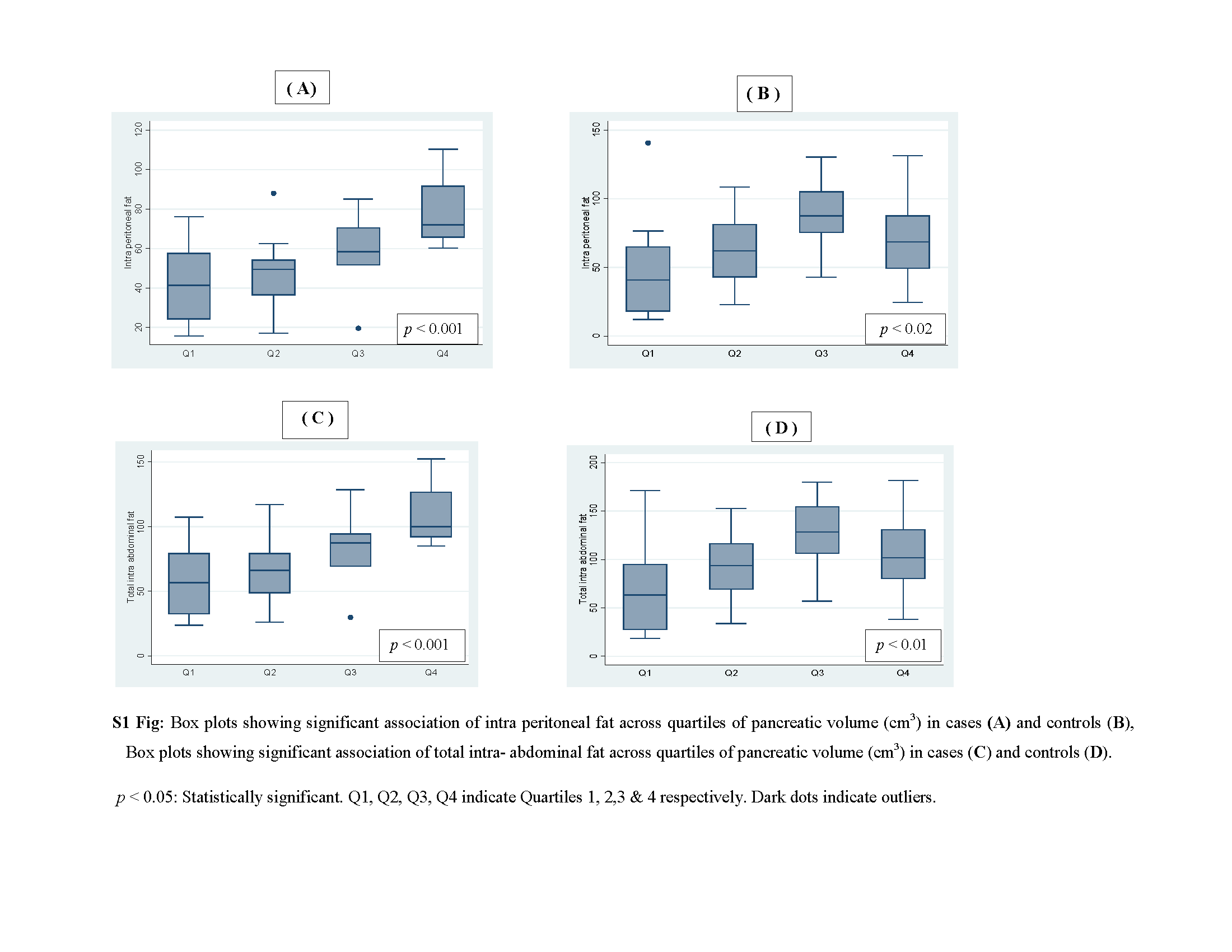

Supplement: S1 Fig — (TIFF) [file pone.0140447.s001.tiff]

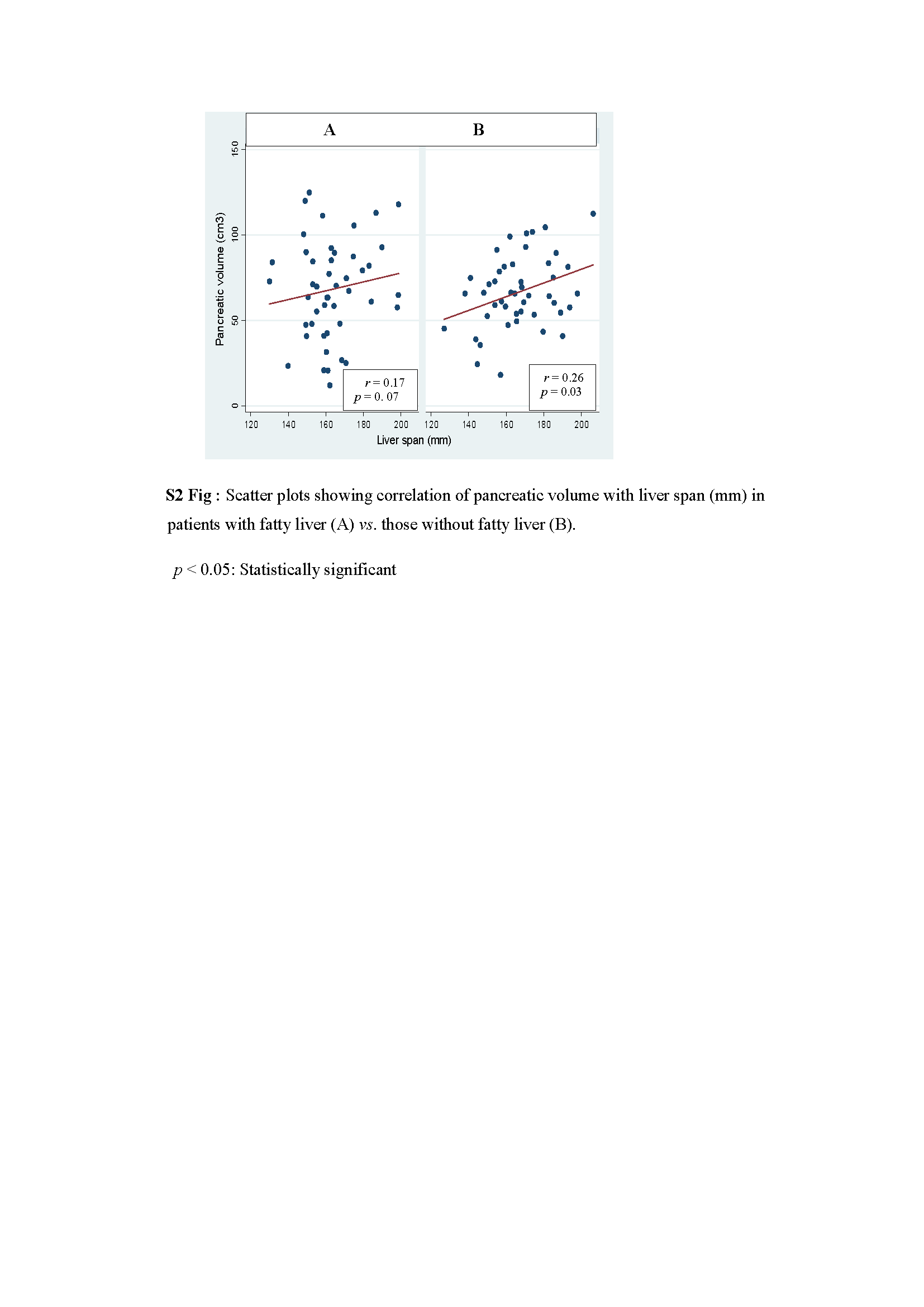

Supplement: S2 Fig — (TIFF) [file pone.0140447.s002.tiff]
