# Supplementary material for: Body Fat Patterning, Hepatic Fat and Pancreatic Volume of Non-Obese Asian Indians with Type 2 Diabetes in North India: A Case-Control Study
Source: PLoS One. 2015 Oct 16;10(10):e0140447. doi: 10.1371/journal.pone.0140447 (PMC4608569; doi:10.1371/journal.pone.0140447)
Supplement: S1 Table — (DOCX) [file pone.0140447.s003.docx]

| Specifications | Abdomen | Liver span | Grading of liver fat | Pancreatic volume |
| --- | --- | --- | --- | --- |
| Pulse sequence | FSPGR Echo | SSFSE | IN/OUT FSPGR | 3D LAVA |
| Scan plane | Oblique axial | Oblique coronal | Oblique axial | Axial |
| Repetition time (TR) (ms) | 135 | Minimum | 100 | 4.2 |
| Echo time TE (ms) | Minimum | 90 | - | - |
| Bandwidth (Khz) | 31.25 | 83.33 | 83.33 | 83.33 |
| Slice thickness (mm) | 8 | 8 | 8 | 4.6 |
| Slice spacing (mm) | 1 | 1 | 2 | 1 |
| Number of slices | 14 | 17 | 21 | 44 |
| Field of view (FOV) | 40 | 40 | 36 | 40 |
| Matrix frequency / phase | 448 / 192 | 320 / 224 | 256 / 224 | 320 / 192 |
| Flip angle | 80 | --- | 80 | 12 |

S1 Table: Technical protocol for quantification of abdominal fat, liver span, liver fat and pancreatic volume using MRI (1.5) Tesla at L2/L3 lumbar vertebrae.

FSPGR: Fast Spoiled Gradient Echo, SSFSE: Single shot Fast Spin Echo, LAVA: Liver acquisition with volume acceleration

FSPGR: Fast Spoiled Gradient Echo, SSFSE: Single shot Fast Spin Echo, LAVA: Liver Acquisition with volume acceleration.
