# Supplementary material for: Body Fat Patterning, Hepatic Fat and Pancreatic Volume of Non-Obese Asian Indians with Type 2 Diabetes in North India: A Case-Control Study
Source: PLoS One. 2015 Oct 16;10(10):e0140447. doi: 10.1371/journal.pone.0140447 (PMC4608569; doi:10.1371/journal.pone.0140447)
Supplement: S2 Table — (DOCX) [file pone.0140447.s004.docx]

**S2 Table**: Comparison of skinfold measurement at eight sites.

| **Skinfold thickness** | **Unadjusted** | | | **Adjusted for age** | | |
| --- | --- | --- | --- | --- | --- | --- |
|  | **Cases**  **(*n* = 93)** | **Controls**  **( *n* = 40)** | ***p* value** | **Cases**  **(*n* = 93)** | **Controls**  **( *n* = 40)** | ***p* value** |
| Biceps (mms) | 8.4 ± 4.1 | 13.6± 9.8 | < 0.01* | 8.3 ± 5.7 | 13.8 ± 6.3 | < 0.01* |
| Triceps (mms) | 15.6 ± 5.3 | 18.9 ± 7.7 | < 0.01* | 15.5 ±0.3 | 19.3 ± 6.3 | < 0.01* |
| Thigh skinfold (mms) | 23.6 ± 5.7 | 28.7 ± 10.1 | < 0.01* | 23.5 ± 6.6 | 28.9 ± 7.5 | < 0.01* |
| Calf skinfold (mms) | 12.4 ± 5.2 | 21.8 ±8.4 | < 0.01* | 12.4± 5.7 | 21.9 ± 6.3 | < 0.01* |
| Subscapular skinfold  (mms) | 22.2 ± 5.8 | 21.8 ± 8.5 | 0.79 | 21.9 ±6.7 | 22.3 ± 6.9 | 0.80 |
| Supra iliac skinfolds (horizontal) (mms) | 17.8 ± 4.9 | 20.7 ± 5.6 | < 0.01* | 17.7 ±4.8 | 20.8 ± 5.0 | < 0.01* |
| Supra iliac skinfolds (vertical) (mms) | 18.1 ± 5.2 | 20.2 ± 5.2 | < 0.05* | 18.0 ±9.6 | 20.4 ± 5.0 | < 0.05* |
| Supra iliac skinfolds (average) (mms) | 17.9 ± 4.8 | 19.9± 5.6 | < 0.05* | 17.8 ±9.6 | 20.1 ± 5.0 | < 0.05* |
| Abdominal skinfolds (vertical) (mms) | 23.9 ± 5.3 | 24.1± 7.8 | 0.87 | 23.8 ±5.7 | 24.6 ± 8.1 | 0.61 |
| Abdominal skinfolds  (horizontal) (mms) | 24.8 ± 5.4 | 24.1 ± 7.6 | 0.60 | 24.8± 5.7 | 24.6 ± 8.1 | 0.91 |
| Abdominal skinfolds  (average) (mms) | 24.2 ± 5.3 | 23.8 ± 7.9 | 0.71 | 24.0± 5.7 | 24.7 ± 8.1 | 0.66 |
| Total peripheral skinfold (mms) | 60.1± 17.3 | 78.0 ± 35.2 | < 0.01* | 59.8 ± 24 | 78.7 ± 23.9 | <0.001* |
| Total truncal skinfolds (mms) | 106 ± 21.8 | 102.0± 29.6 | 0.32 | 106.7±24 | 101.9 ± 25.2 | 0.32 |

Values are presented as Mean ± SD,  *^*^p <* 0.05: Statistically significant.

^#^Total peripheral skinfolds = Sum of biceps, triceps, thigh & calf skinfolds.

^#^Total truncal skinfolds = Sum of sub scapular, supra iliac & abdominal (horizontal &vertical) skinfolds.

^#^Average supra iliac skinfolds = Mean value of supra iliac skinfolds (diagonal + vertical)

^#^Average abdominal skin folds = Mean value of vertical + horizontal abdominal skinfolds
