# Supplementary material for: Body Fat Patterning, Hepatic Fat and Pancreatic Volume of Non-Obese Asian Indians with Type 2 Diabetes in North India: A Case-Control Study
Source: PLoS One. 2015 Oct 16;10(10):e0140447. doi: 10.1371/journal.pone.0140447 (PMC4608569; doi:10.1371/journal.pone.0140447)
Supplement: S3 Table — (DOCX) [file pone.0140447.s005.docx]

**S3 Table:** Body composition as measured by Dual energy X ray absorptiometry.

| **Measures of body composition** | **Unadjusted** | | | **Adjusted for age** | | |
| --- | --- | --- | --- | --- | --- | --- |
|  | **Cases**  **( *n* = 93)** | **Controls**  **(*n* = 40)** | ***p* value** | **Cases**  **( *n* = 93)** | **Controls**  **(*n* = 40)** | ***p* value** |
| Total body fat % | 27.6 ± 6.0 | 30.3 ± 8.3 | < 0.05* | 27.3± 6.7 | 30.9 ± 6.3 | <0.01* |
| Total fat mass(kg) | 17.1 ± 4.9 | 18.0 ± 5.3 | 0.35 | 16.9 ± 4.8 | 18.4 ± 4.4 | 0.12 |
| Total lean muscle mass (kg) | 44.8 ± 5.7 | 40.5 ± 8.4 | < 0.05* | 44.8 ± 6.7 | 41.3 ± 6.3 | < 0.01* |
| Total fat free mass (kg) | 47.3 ± 6.0 | 42.6 ± 9.6 | < 0.01* | 47.4± 7.6 | 42.4 ± 6.9 | < 0.01* |
| Left arm fat % | 23.4 ± 7.4 | 26.4 ± 10.4 | 0.08 | 23.3± 8.6 | 26.8 ± 8.1 | < 0.05* |
| Left arm fat mass (kg) | 1.0 ± 0.7 | 0.9 ± 0.3 | 0.51 | 1.0 ± 0.0 | 0.9± 0.6 | 0.47 |
| Left arm lean mass (kg) | 2.8 ± 0.5 | 2.7 ± 0.8 | 0.17 | 2.8 ± 0.0 | 2.6 ± 0.6 | 0.11 |
| Right arm fat % | 23.5 ± 7.5 | 26.4 ± 10.5 | 0.08 | 23.3± 8.6 | 26.8 ± 8.1 | < 0.05* |
| Right arm fat mass (kg) | 0.9± 0.3 | 0.8 ± 0.3 | 0.65 | 0.9 ± 0.0 | 0.88 ± 0.0 | 0.76 |
| Right arm lean mass (k (kg) | 2.9 ± 0.9 | 2.6 ± 0.9 | 0.08 | 2.9 ± 0.9 | 2.6 ± 0.6 | 0.06 |
| Total leg fat % | 24.1 ± 6.6 | 32.0 ± 10.0 | < 0.001* | 23.9± 7.6 | 32.6 ± 7.5 | < 0.001* |
| Total leg fat mass (kg) | 4.9 ± 1.5 | 7.8 ± 6.1 | < 0.001* | 4.8 ± 3.8 | 8.0 ± 3.1 | < 0.001* |
| Left leg fat % | 24.1 ± 6.4 | 32.1 ± 10.0 | < 0.001* | 23.8 ± 7.6 | 32.6 ± 7.5 | < 0.001* |
| Left leg fat mass (kg) | 2.4 ± 0.7 | 3.5 ± 1.1 | < 0.001* | 2.4 ± 0.0 | 3.5 ± 0.6 | < 0.001* |
| Left leg lean mass (kg) | 7.6 ± 1.2 | 7.6 ± 1.9 | 0.93 | 7.6 ± 0.9 | 7.6 ± 1.2 | 0.81 |
| Right leg fat % | 24.1 ± 6.4 | 32.1 ± 10.0 | < 0.001* | 23.8± 7.6 | 32.7 ± 7.5 | < 0.001* |
| Right leg fat mass (kg) | 2.4 ± 0.7 | 3.4 ± 1.1 | <0.01* | 2.4 ± 0.0 | 3.5 ± 0.6 | < 0.001* |
| Right leg lean mass (kg) | 7.6 ± 1.2 | 7.3 ± 1.4 | 0.19 | 7.7 ± 0.9 | 7.3 ± 1.2 | 0.12 |
| Truncal fat % | 32.1 ± 7.0 | 31.7 ± 8.1 | 0.75 | 31.9 ± 7.2 | 32.3 ± 6.9 | 0.75 |
| Truncal fat mass (kg) | 9.6 ± 2.8 | 8.5 ± 2.8 | < 0.05* | 9.5 ± 2.8 | 8.7 ± 2.5 | 0.14 |
| Truncal lean mass (kg) | 19.7 ± 2.7 | 17.9 ± 3.5 | < 0.01* | 19.7 ± 1.9 | 17.8 ± 2.8 | < 0.001* |

Values are presented as Mean ± SD, *^*^ p <* 0.05*:* Statistically significant.
