# Supplementary material for: Body Fat Patterning, Hepatic Fat and Pancreatic Volume of Non-Obese Asian Indians with Type 2 Diabetes in North India: A Case-Control Study
Source: PLoS One. 2015 Oct 16;10(10):e0140447. doi: 10.1371/journal.pone.0140447 (PMC4608569; doi:10.1371/journal.pone.0140447)
Supplement: S4 Table — (DOCX) [file pone.0140447.s006.docx]

**S4 Table**: Abdominal fat depots, liver span and pancreatic volume, quantified from image obtained by magnetic resonance imaging (1.5 Tesla) at L2/L3 region of the lumbar vertebrae

| **Abdominal fat depots, liver span**  **and pancreatic volume** | **Unadjusted for age** | | | **Adjusted for age** | | |
| --- | --- | --- | --- | --- | --- | --- |
|  | **Cases**  **( *n* = 93)** | **Controls**  **(*n* = 40)** | ***p* value** | **Cases**  **( *n* = 93)** | **Controls**  **( *n* = 40)** | ***p* value** |
| Anterior subcutaneous fat volume (cm^3^) | 49.0 ± 21.7 | 46.7 ± 20.1 | 0.56 | 48.3± 19.9 | 48.4 ± 20.7 | 0.98 |
| Posterior subcutaneous fat volume (cm^3^) | 55.8 ± 20.6 | 57.0 ± 19.4 | 0.75 | 55.2 ± 19.9 | 58.0 ± 20.1 | 0.41 |
| Superficial subcutaneous fat volume (cm^3^) | 80.9 ± 28.7 | 82.4 ± 30.8 | 0.77 | 79.8 ± 28.5 | 84.8 ± 28.9 | 0.35 |
| Deep subcutaneous fat volume (cm^3^) | 22.3 ± 10.5 | 19.6 ± 8.3 | 0.15 | 22.1 ± 9.5 | 20.3 ± 9.4 | 0.35 |
| Total  subcutaneous fat volume (cm^3^) | 103.3 ± 35.3 | 102.1 ± 38.0 | 0.86 | 101.9 ± 35.1 | 105.1 ± 35.2 | 0.64 |
| Retro peritoneal fat volume (cm^3^) | 33.5 ± 14.5 | 19.6 ± 9.0 | < 0.001* | 33.4 ± 12.3 | 19.9 ± 13.2 | < 0.001* |
| Intra-peritoneal fat volume (cm^3^) | 69.3 ± 29.5 | 48.0 ± 22.0 | < 0.001* | 62.2 ± 27.5 | 44.4 ± 27.7 | < 0.001* |
| Total intra- peritoneal fat volume (cm^3^) | 102.8 ± 39.8 | 68.5 ± 30.5 | < 0.001* | 69.1 ± 37.0 | 49.3 ± 37.8 | < 0.001* |
| Liver span (mm) | 165 ± 16.5 | 149.0 ± 19.1 | < 0.001* | 165.1± 17.1 | 149.0 ± 17.6 | < 0.001* |
| Pancreatic volume (cm^3^) | 67.4 ± 24.9 | 53.2 ± 20.8 | < 0.01* | 67.1± 23.7 | 53.9 ± 23.9 | < 0.01* |
| Pancreatic volume index (cm^3^) | 38.7 ± 13.8 | 31.9 ± 11.1 | < 0.01* | 38.6 ±12.3 | 32.2 ± 13.2 | < 0.05* |

Values are presented as Mean ± SD, *^*^ p* < 0.05: Statistically significant.
