# Supplementary material for: Body Fat Patterning, Hepatic Fat and Pancreatic Volume of Non-Obese Asian Indians with Type 2 Diabetes in North India: A Case-Control Study
Source: PLoS One. 2015 Oct 16;10(10):e0140447. doi: 10.1371/journal.pone.0140447 (PMC4608569; doi:10.1371/journal.pone.0140447)
Supplement: S5 Table — (DOCX) [file pone.0140447.s007.docx]

**S5 Table** : Biochemical profile

| **Biochemical variable** | **Unadjusted** | | | **Adjusted for age** | | |
| --- | --- | --- | --- | --- | --- | --- |
|  | **Cases ( *n* = 93)** | **Controls (*n* = 40)** | ***p* value** | **Cases ( *n* = 93)** | **Controls (*n* = 40)** | ***p value*** |
| Fasting blood glucose (mg/dl) | 147.87± 51.13 | 89.55± 8.38 | < 0.01* | 148.0±43.2 | 89.0 ±43.4 | < 0.001* |
| Post prandial blood glucose (mg/dl) | 222.59 ± 82.92 | 89.36 ± 13.69 | < 0.01* | 222.9 ±70.5 | 87.6±67.2 | < 0.01* |
| Glycosylated haemoglobin (%) | 9.00 ± 2.49 | 5.19 ± 0.37 | < 0.01* | 9.0±1.9 | 5.1 ± 1.9 | < 0.001* |
| Total cholesterol (mg/dl) | 175.44± 41.50 | 152.48 ±29.14 | < 0.01* | 175.4±38.4 | 152.6 ± 42.2 | 0.34 |
| Triglycerides (mg/dl) | 170.10 ± 99.47 | 97.15± 45.88 | < 0.01* | 170.0±87.3 | 97.4±88.2 | < 0.001* |
| High density lipoprotein cholesterol (mg/dl) | 40.77 ± 8.93 | 42.47 ± 8.33 | 0.31 | 40.8 ±8.6 | 42.4 ±8.1 | < 0.05* |
| Low density lipoprotein cholesterol (mg/ dl) | 110.36 ± 31.27 | 96.81± 25.32 | < 0.05* | 110.2 ±29.7 | 98.0 ±29.6 | < 0.001* |
| Very low density lipoprotein cholesterol (mg/dl) | 33.5± 19.32 | 19.47± 9.18 | < 0.01* | 19.5 ±16.3 | 33.5 ±17.0 | < 0.001* |
| Serum glutamic pyruvic transaminase (U/L) | 57.81± 21.76 | 45.60± 18.12 | < 0.01* | 57.9 ±20.1 | 47.1±21.4 | 0.17 |
| Serum glutamic oxaloacetic transaminase (U/L) | 27.89 ± 12.84 | 23.68 ± 9.18 | < 0.05* | 27.8 ±11.5 | 24.6 ±11.9 | < 0.001* |

Values are presented as Mean ± SD,  *^*^p <* 0.05: Statistically significant.
