# Supplementary material for: Body Fat Patterning, Hepatic Fat and Pancreatic Volume of Non-Obese Asian Indians with Type 2 Diabetes in North India: A Case-Control Study
Source: PLoS One. 2015 Oct 16;10(10):e0140447. doi: 10.1371/journal.pone.0140447 (PMC4608569; doi:10.1371/journal.pone.0140447)
Supplement: S6 Table — (DOCX) [file pone.0140447.s008.docx]

| Cases (*n* = 93) | | | | | | Controls (*n* = 40) | | | | |
| --- | --- | --- | --- | --- | --- | --- | --- | --- | --- | --- |
| Abdominal fat depots and liver span measured by MRI(1.5 ) Tesla at L2/L3 lumbar vertebrae | Quartile 1  ( *n* = 14) | Quartile2  ( *n* = 23) | Quartile 3  ( *n* = 27) | Quartile4  (*n* = 29) | *p* value | Quartile1  ( *n* = 19) | Quartile 2  ( *n* = 11) | Quartile3  ( *n* =6 ) | Quartile 4  ( *n* = 4) | *p*  value |
| Anterior subcutaneous fat volume (cm^3^) | 36.7  (10.8 – 84.7) | 47.2  (23.3 -171.2) | 50.7  (18.7-85.8) | 52.1  (26.3–69.7) | 0.06 | 35.3  (13.7-73.3) | 49.6  (27.1 - 90.3) | 38  (15.2 -52) | 51.6  (32.3 - 89.8) | 0.20 |
| Posterior subcutaneous fat volume (cm^3^) | 48.4  (17.4- 88.2) | 54.2  (24.7 -108.7) | 61.2  (32.2-101.8) | 56.1  (26.6-101.9) | 0.26 | 51.3  (26.1- 100) | 60.0  (29.2 - 88.4) | 46.1  (24- 69.4) | 68.0  (55.4- 73.8) | 0.29 |
| Superficial subcutaneous fat volume (cm^3^) | 56.2  (23.2 -129) | 89.4  (37.1 -156.6) | 90.1  (42 - 131.4) | 79  (46.8 -128.2) | 0.14 | 68.1  (36.2- 136) | 82.6  (30 - 147.6) | 68.6  (31.8- 101.5) | 89.2  (80.1- 123) | 0.30 |
| Deep subcutaneous fat volume (cm^3^) | 16  (5.3 - 41) | 21  (11. 0 - 47) | 23.3  (8.2 -53.7) | 20.7  (8.6 -65.4) | 0.14 | 16.3  (7.1 - 37.3) | 19.0  (6.6 - 32.3) | 15.2  (8.2 - 20.2) | 25.4  (19.1-39.1) | 0.12 |
| Retroperitoneal fat volume (cm^3^) | 22.4  (4.6 - 56) | 29.8  (11.0 -85.1) | 34.4  (13.4 - 59.6) | 40  (14 - 60.4) | <0.05^*^ | 15.8  (7.2 -31.2) | 16.9  (8.8- 29.1) | 25.5  (10.2- 43.5) | 27.9  (24.8 - 42.1) | < 0.05^*^ |
| Intra- peritoneal fat  volume(cm^3^) | 40.8  (12.2 - 140.6) | 62.1  (22.8 -108.6 | 68.7  (24.5-131.5) | 87.6  (42.8- 130.4) | < 0.01^*^ | 41.3  (15.6 – 76.2) | 49.4  (17.1 -88) | 58.3  (19.5 -85) | 72.0  (60.2 -110.3) | < 0.05^*^ |
| Total intra-abdominal fat volume(cm^3^) | 63.2  (18.4 -171.2) | 93.7  (33.8 -152.4) | 101.6  (38.2 -181.4) | 128.2  (56.8 -179.6) | < 0.01^*^ | 56.7  (23.7 -107.4) | 66.0  (26.0 -117.1) | 87.4  (29.7 -128.5) | 100  (85.1 -152.4) | < 0.05^*^ |
| Liver span (mm) | 66  (23.3 -120) | 62.4  (18.1 -124.9) | 63.4  (12- 99.2) | 75.1  (40.8 - 118) | 0.08  (NS) | 49.4  (16.9 -123.6) | 47.3  (30.4- 80.4) | 41.9  (27.2- 70.5) | 59.6  (36.6 -109.3) | 0.71 |

**S6Table:** Association of abdominal fat depots and liver span across quartiles of pancreatic volume.

Total intra-abdominal fat; cases: Q1 *vs* Q2: *p* < 0.05, Q1 *vs* Q3: *p* < 0.001,Q1 *vs.* Q4: *p <* 0.01, Q2 *vs.* Q3: *p <* 0.01, Q3 *vs* Q4: *p* < 0.05

Total intra-abdominal fat; controls: Q1 *vs* Q4: *p <*0.01, Q2 *vs* Q4: *p* < 0.01

Retroperitoneal fat; cases: Q1 *vs* Q3: *p* < 0.001, Q1 *vs* Q4:*p <* 0.01, Q2 *vs* Q3:*p <* 0.05, controls: Q1 *vs.* Q4:*p <* 0.01

Intra-peritoneal fat; cases: Q1 *vs* Q2: *p* < 0.05, Q1 *vs* Q3:*p* <0.001, Q1 *vs* Q4:*p <* 0.001, Q2 *vs* Q3:*p <*0.001, Q3 *vs* Q4:*p* < 0.05

Intra- peritoneal fat; controls: Q1 *vs* Q4: *p <* 0.05, Q2 *vs* Q4:*p* < 0.05

Liver span; cases: Q3 *vs* Q4: *p* < 0.05

Values are presented as median (minimum - maximum),*^*^p* < 0.05: Statistically significant.
